# Basic emotion recognition of children on the autism spectrum is enhanced in music and typical for faces and voices

**DOI:** 10.1371/journal.pone.0279002

**Published:** 2023-01-11

**Authors:** Shalini Sivathasan, Hadas Dahary, Jacob A. Burack, Eve-Marie Quintin

**Affiliations:** 1 Department of Educational and Counselling Psychology, McGill University, Montreal, Quebec, Canada; 2 Azrieli Centre for Autism Research, Montreal Neurological Institute, McGill University, Montreal, Quebec, Canada; 3 Center for Research on Music, Brain, and Language, McGill University, Montreal, Quebec, Canada; Istituto di Fisiologia Clinica Consiglio Nazionale delle Ricerche, ITALY

## Abstract

In contrast with findings of reduced facial and vocal emotional recognition (ER) accuracy, children on the autism spectrum (AS) demonstrate comparable ER skills to those of typically-developing (TD) children using music. To understand the specificity of purported ER differences, the goal of this study was to examine ER from music compared with faces and voices among children on the AS and TD children. Twenty-five children on the AS and 23 TD children (6–13 years) completed an ER task, using categorical (happy, sad, fear) and dimensional (valence, arousal) ratings, of emotions presented via music, faces, or voices. Compared to the TD group, the AS group showed a relative ER strength from music, and comparable performance from faces and voices. Although both groups demonstrated greater vocal ER accuracy, the children on the AS performed equally well with music and faces, whereas the TD children performed better with faces than with music. Both groups performed comparably with dimensional ratings, except for greater variability by the children on the AS in valence ratings for happy emotions. These findings highlight a need to re-examine ER of children on the AS, and to consider how facilitating strengths-based approaches can re-shape our thinking about and support for persons on the AS.

## Introduction

Differing patterns of emotion recognition (ER) have long been cited as significant components of the socialization profile that is characteristic of persons on the autism spectrum (AS) as compared “typically-developing” (TD), persons [1–7]. However, virtually all of the evidence of relative ER challenges or “deficits” has been based on findings with paradigms in which the presentation of emotions occurs in the context of socially explicit facial and vocal stimuli. This evidence of reduced ER accuracy diverges from that of comparable abilities between persons on the AS and TD persons to recognize emotions when the tasks are presented in the context of music [8–10], a domain in which persons on the AS have been found to display a variety of unique abilities [e.g., 11–13]. To better understand the nature of these disparate findings, we investigated ER skills with musical, as compared to facial and vocal, stimuli using both categorical and dimensional response options in order both to provide a nuanced understanding of ER processing across various types of stimuli and to consider the ways that strength-based approaches can re-shape our thinking about persons on the AS.

Music is a stimulus with which persons on the AS regularly and readily engage in their day-to-day lives, and with which they have demonstrated a variety of skills and strengths [13]. Persons on the AS have been found to show enhanced pitch discrimination of musical tones and melodies [14–18] and musical memory [19–21] as compared to mental age (MA)-matched TD persons. Among children on the AS, verbal production may be enhanced through the incorporation of music into speech- and language-focused interventions, particularly for children who have greater language and learning needs [e.g., 22–25]. Further, music therapy has been shown to improve social interaction and communication skills, such as joint attention [26, 27], turn taking [26], and social communication [28] when the development of these skills is the target of the intervention.

The study of ER skills of persons on the AS involves a similar strengths-based approach via the inclusion of musical stimuli. For example, Heaton et al. [8] found that children on the AS show comparable abilities to MA-matched TD children in distinguishing music conveying happy (typically in a major musical scale) and sad (in a minor scale) emotions. Concordantly, comparable ER ratings of happy, sad, and fearful musical excerpts between children and adolescents on the AS and their TD peers have been found when differences in verbal IQ were controlled [9, 10]. Similar performance on behavioral ER tasks as well as on those involving the activation of emotion processing and reward neurocircuitry when listening to happy and sad music have been shown between persons on the AS and TD persons [29, 30]. The evidence from these musical ER studies suggests that persons on the AS appear to be able to recognize basic emotions conveyed by music comparably to their TD peers of a similar developmental level. In contrast, the findings from meta-analyses of behavioral ER studies indicate medium to large overall effect sizes across studies indicating lower accuracy levels on recognition tasks of basic emotions among adults and children on the AS as compared to TD participants when socially explicit stimuli (e.g., faces, voices) were used [6, 7, 31]. This suggests a discrepancy between findings based on socially explicit stimuli versus less socially explicit stimuli that can also convey emotions (i.e., music).

Attempts to reconcile such discrepancies need to address how music differs in its conveyance of emotion as compared to explicitly social facial and vocal stimuli. Emotions are most commonly presented in an interpersonal or self-reflective context, such as through social observation and interaction or through an intrapersonal emotional experience. This use of socially explicit facial and vocal stimuli in ER studies with persons on the AS may contribute to the finding of reduced ER accuracy on behavioral tasks. For example, differences in facial ER accuracy among persons on the AS relative to TD persons could be related to early developmental differences in reduced attention to eyes and faces [32] or to the reduced value and salience of social versus non-social stimuli that begins in childhood [33, 34]. In contrast, music can elicit an affective reaction in the listener without requiring a need to understand or empathize with the composer’s mental states or emotional intentions [8, 35]. Emotions are conveyed in music through variations in structural auditory aspects such as tempo, timbre, pitch, and mode, and thereby afford a unique yet common and familiar vehicle for emotional experience [36]. Accordingly, the primary goal of the current study is to reconcile reported differences in ER among children on the AS, and specifically to determine whether music affords a relative ER strength in comparison with explicitly social (i.e., facial and vocal) stimuli, and whether such a pattern of strengths and challenges is observed among their TD peers. If children on the AS demonstrate relative ER strengths using less socially explicit stimuli and TD children demonstrate relative ER strengths using more socially explicit stimuli, we could speculate that differing routes for understanding and teaching ER skills are available to children with diverse strengths and needs.

The disparities in findings across studies may also be due to the differences in the measurement techniques that are used in experimental designs and methodologies in ER research [e.g., 7, 37–40]. Based on the theory that basic emotions are universally expressed and recognized [41, 42], ER abilities are frequently measured with behavioral paradigms using these discrete categorical representations (e.g., happy, sad, fear) through a forced-choice response format with verbal labels. However, emotional expressions can also be considered a product of dimensional attributes, particularly valence (positive to negative) and arousal (high to low energy) [43], and therefore can also be measured using continuous ratings [43, 44; 36 for a review]. While some of the earliest experimental studies of ER among persons on the AS have involved a categorical approach [e.g., 4, 5], the use of dimensional methods is becoming increasingly more common [e.g., 29, 45]. Accordingly, in order to capture dynamic aspects of emotions across each domain along a continuum of valence and arousal ratings, our second goal was to provide a direct comparison of musical, facial, and vocal ER among children on the AS and TD children with dimensional ratings versus traditional categorical response options.

### Objectives and hypotheses

The main objective of this study was to compare the processing of basic emotions using musical, facial, and vocal stimuli among children on the AS and TD children. In comparing the groups, we hypothesized that the AS group would demonstrate a relative strength in musical ER compared to the TD group, whereas the TD group might display a strength in ER with socially explicit stimuli (faces, voices) relative to the AS group. We also hypothesized that there would be discrepancies in ER with a relative weakness in facial and vocal ER as compared to musical ER within the AS group, whereas we did not expect any significant difference in performance among musical, facial, and vocal ER within the TD group.

We also explored the use of dimensional ratings of valence and arousal across the musical, facial, and vocal stimulus types. As this aim was exploratory, we did not venture specific hypotheses regarding between or within group differences. To allow for a direct comparison of ER across the three domains, we developed an integrated experimental task displaying the musical, facial, and vocal stimuli, with categorical and dimensional response options presented for each item presented. We focused on comparisons from the three basic emotions of happy, sad, and fear that can be reliably conveyed by music [8–10].

## Method

### Participants

The initial group of participants included 29 children on the AS recruited from special education schools or classrooms and 24 TD children recruited from schools or the general community in Quebec, Canada. The data from five participants (4 AS and 1 TD) were excluded from the analyses because one participant on the AS did not complete the verbal comprehension portion of the cognitive test and the scores on the experimental task of the other four participants were multivariate outliers. Accordingly, the final group of participants (*N* = 48) included 25 children on the AS (19 boys) aged 9–13 years (*M* = 11 years) and 23 TD children (11 boys) aged 6–12 years (*M* = 9.7 years).

All of the participants on the AS had an educational code of autism spectrum disorder derived from expert diagnoses from pediatricians, child psychiatrists, or psychologists (for a discussion of the educational code system in Quebec, see [46, 47]). The Parent and Teacher versions of the Social Responsiveness Scale, 2^nd^ edition (SRS-2 [48]), were also used to ascertain the presence or absence of autism traits among the AS and TD groups, respectively. The average SRS Total T-Score was greater than the clinical cut-off of 60 for the AS group and below 60 for the TD group. Four participants on the AS had T-Scores slightly below (between 54–59) the cutoff and three TD participants had scores at or above (60–62). As a similar pattern of results were found when the analyses were run with and without these seven participants with the categorical response option, the full dataset was used in the reported analyses.

The verbal scales of the Wechsler Intelligence Scale for Children, Fifth Edition (WISC-V [49]) in English or French, or the Wechsler Abbreviated Intelligence Scale, Second Edition (WASI-II [50]) in English (no French version available) were used to estimate cognitive skills, and scores obtained were derived from the Verbal Comprehension Index (VCI) and Full-Scale IQ. Consistent with recommendations to consider the effects of verbal cognitive ability on ER task performance, and to account for variability in VCI and age ranges between groups [51–53], verbal mental age (VMA) was calculated using the participants’ VCI and chronological age (AS group VMA: *M* = 8.48 [5–11 years]; TD group VMA: *M* = 10.03 [5–15 years]).

The AS and TD groups differed significantly in terms of SRS-2 Total Scores, chronological age, IQ, and VMA (Table 1). Thus, VMA was considered as a covariate in our analyses of the group comparisons. Maternal educational attainment levels and annual income reported by 75% of participants’ caregivers did not differ significantly between groups (see S1 Table). Specific demographic data on race & ethnicity were not systematically collected during this study and were, therefore, not available for the analyses.

**Table 1:** Participant characteristics of the AS and TD groups.

	AS Group (*n* = 25)			TD Group (*n* = 23)			*p*
	M	SD	Range	M	SD	Range	
Chronological Age	11.02	1.12	9–13	9.70	1.63	6–12	.003
Verbal Mental Age	8.48	1.62	5–11	10.03	2.56	5–15	.018
VCI	77	14	51–104	102	14	73–128	< .0001
FSIQ^a^	79	14	54–109	104	13	78–124	< .0001
SRS-2	71	9	54–88	49	8	40–62	< .0001

*Note*. Mean (M), standard deviation (SD), ranges, and *p* values of an independent sample t-test between the AS group and the TD group. Chronological age (CA), and Verbal Mental Age (VMA) are displayed in years. Verbal Comprehension Index (VCI) and Full-Scale IQ (FSIQ) = standard score. Social Responsiveness Scale-2 (SRS-2) Total Score = T-Score.

^a^AS group *n* = 24, TD group *n* = 22.

### Experimental task

#### Musical, facial, and vocal stimuli

The ER task included 72 different musical, facial and vocal stimuli depicting emotions of happy, sad, or fear. The task was divided into 3 blocks (one per stimulus type: music, faces, voices), with each block containing 24 items (8 happy, 8 sad, and 8 fear). Each item within a block was presented briefly on the computer screen for 1.5–2 seconds. The vocal and musical stimuli selected from the validated sets described below were purposefully designed to be short emotional “bursts” representing the minimum basic emotional information required to appraise the emotion without providing extra semantic (or any linguistic) content [54, 55]. Selected facial stimuli were thus also presented at a short duration to match that of the auditory stimuli, as well as to mitigate against potentially making the task too easy by providing longer stimulus presentations [37]. The order of the three stimulus types (music, faces, voices) was counterbalanced across the participants and the order of the items within each stimulus block was randomized.

*Faces*. The participants viewed 24 color photos of closed-mouth faces (happy, sad, or fear) selected from the NimStim Set of Facial Expressions [56], a well-validated set of photos of facial expressions conveying basic emotions. The photos were of Asian, Black, Latin-American, and White adults, providing a racially and ethnically diverse sample. In total, 8 unique adult actors (*n* = 4 women) represented 3 emotions (3 emotions x 2 sexes x 4 ethnicities = 24 faces). Each selected facial expression had a reliability coefficient of.78 or greater, based on validation with TD adults [54].

*Voices*. The participants listened to 24 selected vocal sounds from the Montreal Affective Voices battery (MAV [54]), a validated set of nonverbal emotional vocalizations (e.g., laugh = happy, cry = sad, scream = fear) produced by 10 actors (*n* = 5 women). Each actor’s vocalization was validated by TD adults, producing a reliability coefficient of.67 or greater.

*Music*. The participants listened to 24 short instrumental melodies or succession of chords played on a violin, from the Musical Emotional Bursts battery (MEB [55]), a validated set of musical clips designed as a musical counterpart to the MAV vocal stimuli. Each selected clip that conveyed happiness, sadness, or fear had a reliability coefficient of.70 or greater validated with TD adults.

#### Categorical and dimensional response conditions

A three-tiered response system (a categorical condition and a two-part dimensional condition) was provided following the presentation of each stimulus item within the three blocks. The order of response conditions was counterbalanced across the participants, with half of the participants (*n* = 24) responding with the categorical condition first and the other half with the dimensional condition first.

**Fig 1:**
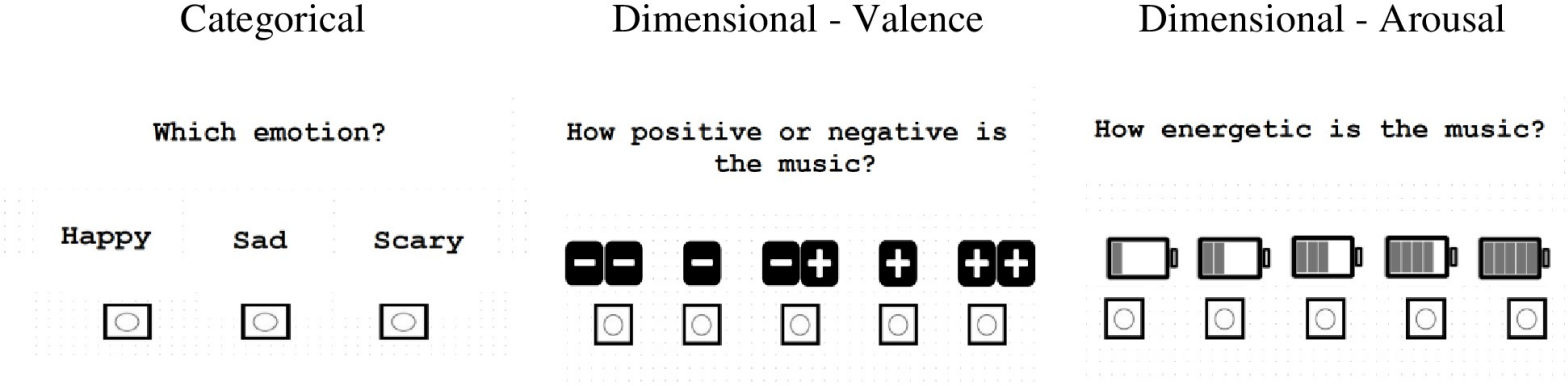
Example categorical and dimensional response options.

*Categorical condition*. In the categorical response condition, each item was presented and then followed by the on-screen instruction, “Which emotion?” with three verbal labels of happy, sad, and fear (the word scared was used when depicting emotions evoked through faces and voices, and scary for emotions evoked through music; see Fig 1). The participants selected their response by clicking on one of the three options using a mouse. Accuracy of emotion recognition was determined by the percentage of correct responses per emotion and also per stimulus type.

*Dimensional condition*. In the dimensional response condition, the presentation of each item was followed by the on-screen instruction, “How positive or negative is the face (or voice or music)?” The participants provided a valence rating on a 5-point Likert scale (from very negative to very positive). They were also asked “How energetic is the face (or voice or music)?” to which they provided an arousal rating on a 5-point Likert scale (from very low energy to very high energy). In order to minimize the verbal demand of the task and to make the task more child-friendly, dimensional Likert scale anchors were paired with cartoon icons. Addition and subtraction symbols were used for the valence scale and batteries with varying levels of energy were used for the arousal scale (see Fig 1).

### Procedure

All of the procedures for this study were approved by McGill University’s Research Ethics Board (#101–0716). As Quebec is a bilingual (English and French) province, all of the materials and procedures were available or translated into both languages and administered in the participant’s dominant language by bilingual clinically trained research staff. The participants were tested in a research lab at the university or in the child’s school or home. Written informed consent was obtained from the parents and written or verbal assent was provided by each participant prior to the beginning of the study.

All of the participants successfully completed a basic hearing test at www.legroupeforget.com. The hearing test and experimental task were administered on a PC laptop with over-ear headphones. The experimental task was completed in the E-prime (version 3.0) environment using a laptop and mouse. In order to ensure the comprehension of both response conditions, a practice session was administered first. All of the instructions were available on the screen in the participants’ dominant language and were read to them during the practice session. A more extensive explanation of the Likert scales was provided during the practice session if needed. The experimental task took approximately 20 minutes to complete.

### Analysis plan

Separate repeated measures analyses of covariance (ANCOVAs) were run for each of the categorical, dimensional valence, and dimensional arousal conditions, to understand the effects of group (AS, TD), stimulus type (music, face, voice), and emotion (happy, sad, fear), while controlling for VMA. Significant effects were further examined in two ways: 1) between-group comparisons, using two-way repeated measures ANOVAs to examine group differences in task performance for each stimulus type and/or emotion, while controlling for differences in VMA of all the participants; and 2) within-group comparisons, using repeated measures ANOVAs to examine differences in task performance across stimulus types and/or emotions within each group, controlling for VMA for each group. To yield medium-sized effects (F = .25) with 80% power and alpha at.05, a priori power analyses using G*Power 3.1 indicated that a total sample size of 46 participants would be needed. Bonferroni corrections for multiple comparisons were applied when appropriate. The task performance means and means adjusted for VMA are presented in Tables 2 and 3.

**Table 2:** Means, adjusted means, standard deviations and standard errors for categorical ER accuracy ratings.

	AS Group		TD Group		F	η_p_^2^
	Mean (SD)	Adjusted Mean (SE)	Mean (SD)	Adjusted Mean (SE)		
Stimulus Type					6.58*	.13
Music	.78 (.14)	.80 (.03)	.70 (.18)	.69 (.03)	5.40*	.11
Face	.80 (.14)	.81 (.01)	.85 (.09)	.84 (.01)	.94	.02
Voice	.92 (.07)	.92 (.03)	.91 (.06)	.91 (.03)	.22	.005
Emotion					3.05	.06
Happy	.89 (.13)	.90 (.02)	.87 (.09)	.85 (.02)		
Sad	.81 (.14)	.82 (.03)	.87 (.11)	.86 (.03)		
Fear	.80 (.14)	.81 (.03)	.74 (.18)	.73 (.03)		

*Note*. Means adjusted for VMA of the overall sample = 9.22 years. Statistics for individual emotions are not reported because the emotion by group interaction effect was not significant.

*p < .05

**Table 3:** Means, adjusted means, standard deviations and standard errors for dimensional ratings of valence and arousal.

	AS Group		TD Group		F	η_p_^2^
	Mean (SD)	Adjusted Mean (SE)	Mean (SD)	Adjusted Mean (SE)		
Valence Ratings						
Stimulus Type					1.55	.03
Music	3.00 (.57)	2.97 (.10)	2.80 (.37)	2.83 (.10)		
Face	2.88 (.35)	2.87 (.07)	2.93 (.31)	2.94 (.07)		
Voice	2.93 (.46)	2.93 (.09)	2.84 (.37)	2.85 (.09)		
Emotion					4.30*	.09
Happy	4.32 (.34)	4.33 (.09)	3.91 (.47)	3.90 (.09)	11.77*	.21
Sad	2.12 (.60)	2.09 (.11)	2.19 (.50)	2.22 (.12)	.57	.01
Fear	2.37 (.75)	2.34 (.14)	2.47 (.54)	2.50 (.14)	.66	.01
Arousal Ratings						
Stimulus Type					2.47	.05
Music	3.20 (.46)	3.26 (.10)	3.15 (.59)	3.08 (.11)		
Face	3.03 (.35)	3.04 (.08)	3.15 (.42)	3.14 (.09)		
Voice	3.20 (.60)	3.23 (.12)	3.22 (.53)	3.18 (.12)		
Emotion					1.87	.04
Happy	4.15 (.48)	4.12 (.10)	3.81(.50)	3.84 (.11)		
Sad	2.41 (.59)	2.44 (.12)	2.65 (.56)	2.62 (.13)		
Fear	2.87 (.86)	2.97 (.16)	3.04 (.77)	2.94 (.17)		

Means adjusted for VMA of the overall sample = 9.22 years. Statistics for individual emotions and stimulus types are not reported when corresponding group by stimulus type or group by emotion interactions are not significant.

*p < .05

## Results

### Categorical response condition

**Fig 2:**
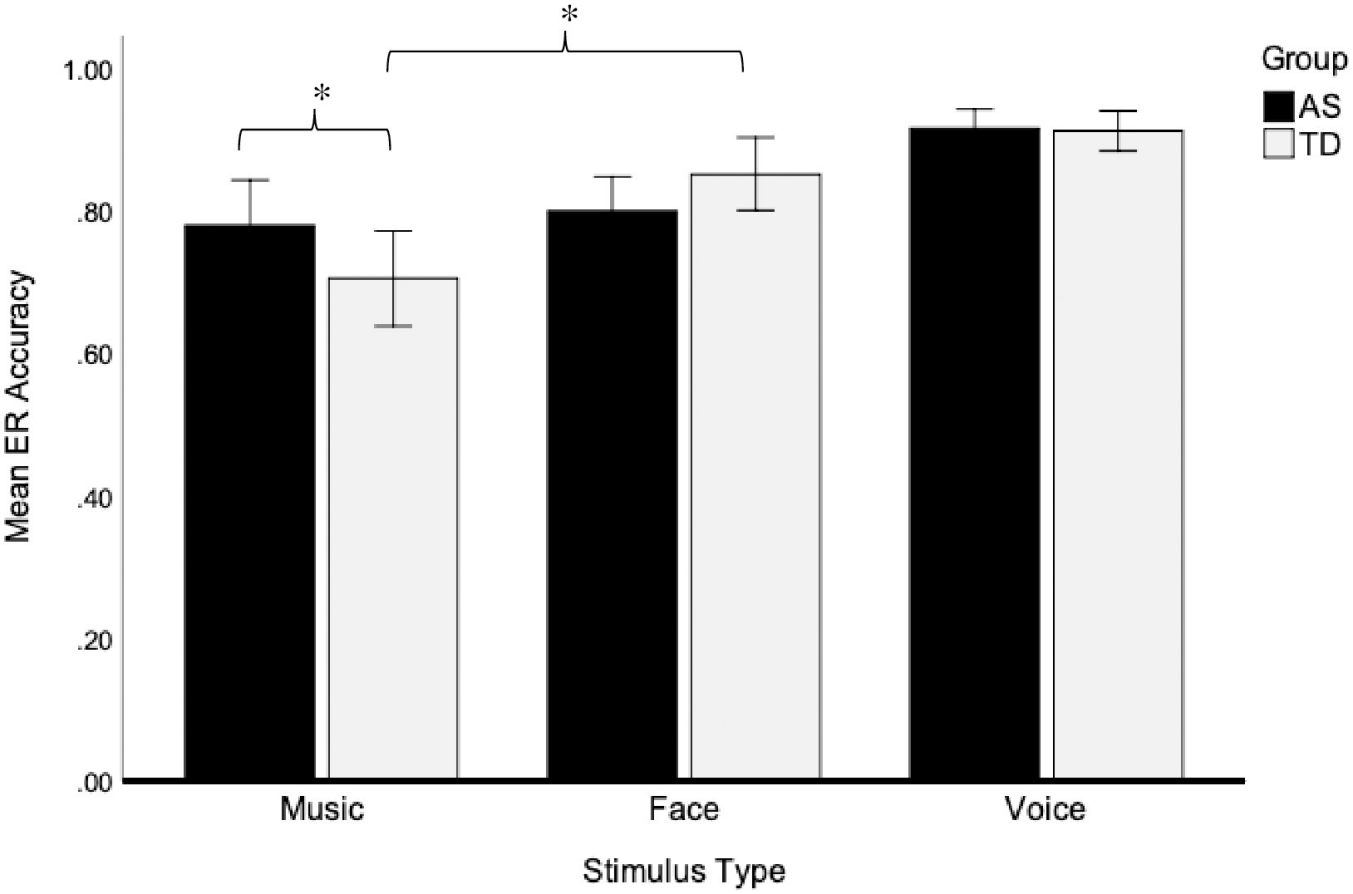
Between- and within-group differences across stimulus types in the categorical condition. Musical emotion recognition (ER) accuracy was significantly higher among the children on the AS as compared to the TD children, whereas the groups did not differ in ER from voices or faces. Although both groups had higher ER accuracy from voices, the TD group had significantly lower ER accuracy from music than faces whereas no differences emerged between music and faces for the AS group. Means and standard error bars are shown. *p < .05.

The three-way interaction among group, stimulus type, and emotion on categorical ER accuracy ratings was not statistically significant, F(2.98, 134.22) = 2.15, *p* = .098, η_p_^2^ = .045. A significant main effect of stimulus, F(1.61, 72.24) = 7.48, *p* = .002, η_p_^2^ = .14, and a two-way interaction between group and stimulus, F(1.61, 72.24) = 6.58, *p* = .004, η_p_^2^ = .13, were found when controlling for VMA (see Table 2 and Fig 2). Follow up analyses revealed a significant between-group difference in ER accuracy within the musical stimuli, F(1,45) = 5.40, *p* = .025, η_p_^2^ = .11, as the AS group had higher mean accuracy than the TD group when recognizing emotions presented in music. In contrast, no differences were found between the AS and TD groups on ER accuracy within the vocal stimuli, F(1, 45) = .22, *p* = .64, η_p_^2^ = .005, or the facial stimuli, F(1, 45) = .94, *p* = .34, η_p_^2^ = .02, suggesting that the children in the AS and TD groups performed equally well in terms of ER accuracy from voices and faces.

The two-way interaction between group and stimulus type was also explored by comparing ER accuracy ratings across the three stimulus types (music, face, voice) within each group. The main effect of stimulus type was significant within the TD group, F(1.31, 27.43) = 6.98, *p* = .009, η_p_^2^ = .25, but not within the AS group, F(2, 46) = 1.09, *p* = .34, η_p_^2^ = .045. Nevertheless, given our a priori hypothesis, we compared responses across stimulus types within both groups. In both groups, ER accuracy was highest for emotions from voices (AS group: *M* = .92, TD group: *M* = .91), *ps* ≤.001. The children in the TD group recognized emotions more accurately from faces compared to music, *p* < .001, whereas no significant difference was found between ER accuracy from faces compared to music for the AS group, *p* = .36.

Neither the main effects of group and emotion nor the interaction effects of emotion by stimulus type and emotion by group were significant (all *p* values >.05), suggesting that happy, sad, and fear emotions were equally well identified within and between the AS and TD groups, and across the stimulus types. VMA was not a significant covariate in the model, and neither the interaction effects of VMA by emotion nor of VMA by stimulus type were significant (all *p* values >.05).

These results support our hypothesis that children on the AS show a strength in identifying emotions within the musical stimuli compared to TD children. However, contrary to another hypothesis, the TD children did not demonstrate a strength in ER from facial and vocal stimuli as compared to the children on the AS. Our within group hypotheses were also not supported. Specifically, the children on the AS did not demonstrate a relative weakness in facial or vocal ER as compared to musical ER. Both groups had better ER accuracy from voices, and the children on the AS performed comparably between faces and music, whereas the TD children performed better with faces than music. Overall, the children on the AS showed a strength in recognizing emotions in music in comparison to the TD children, but not in comparison to their ability to recognize emotions in faces and voices.

### Dimensional response condition

#### Valence ratings

**Fig 3:**
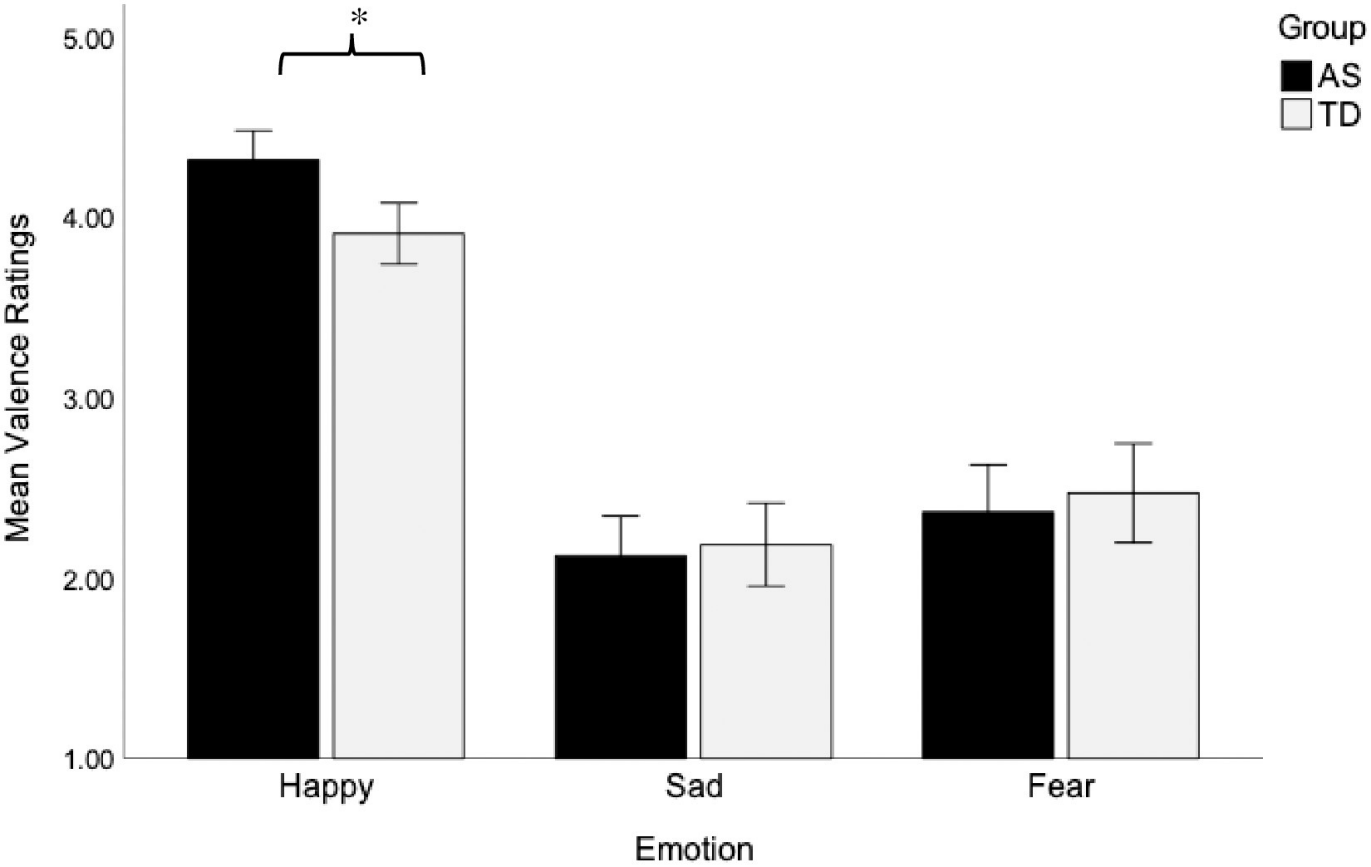
Between-group difference in valence ratings for happy emotions. The children on the AS rated happy stimuli as more positive than did the TD children, whereas the groups did not differ in valence ratings of sad and fear emotions. Means and standard error bars are shown. *p = .001.

A three-way interaction among group, stimulus type, and emotion on valence ratings was not statistically significant, F(4, 180) = .86, *p* = .49, η_p_^2^ = .02. Neither a significant main effect of emotion nor a significant two-way interaction between group and emotion, F(1.52, 68.35) = 4.30, *p* = .026, η_p_^2^ = .09, were found while controlling for VMA. Follow up analyses revealed a significant group difference in valence ratings for happy emotions, F(1, 45) = 11.77, *p* = .001, η_p_^2^ = .21, such that the AS group rated happy emotions as significantly more positive as compared to the TD group. In contrast, no differences were found between the AS and TD groups on mean valence ratings of sad, F(1, 45) = .57, *p* = .46, η_p_^2^ = .01, or fear emotions, F(1, 45) = .66, *p* = .42, η_p_^2^ = .01, suggesting they rated emotional valence for these two negative emotions similarly (see Table 3 and Fig 3).

The main effects of group and stimulus type, and the interaction effects of stimulus type by group and stimulus type by emotion were not significant (all *p* values >.05), suggesting that the valence of emotions for both groups was equally well identified regardless of the stimuli in which they were presented. VMA was not a significant covariate in the model, and the interaction effects between VMA and emotion and stimulus type were not significant (all *p* values >.05).

These results suggested that both groups rated the valence of emotions similarly (i.e., happy emotions as more positive than sad and fear), regardless of the stimuli in which they were presented, although the children on the AS rated happy emotions as more positive than did the TD children.

**Fig 4:**
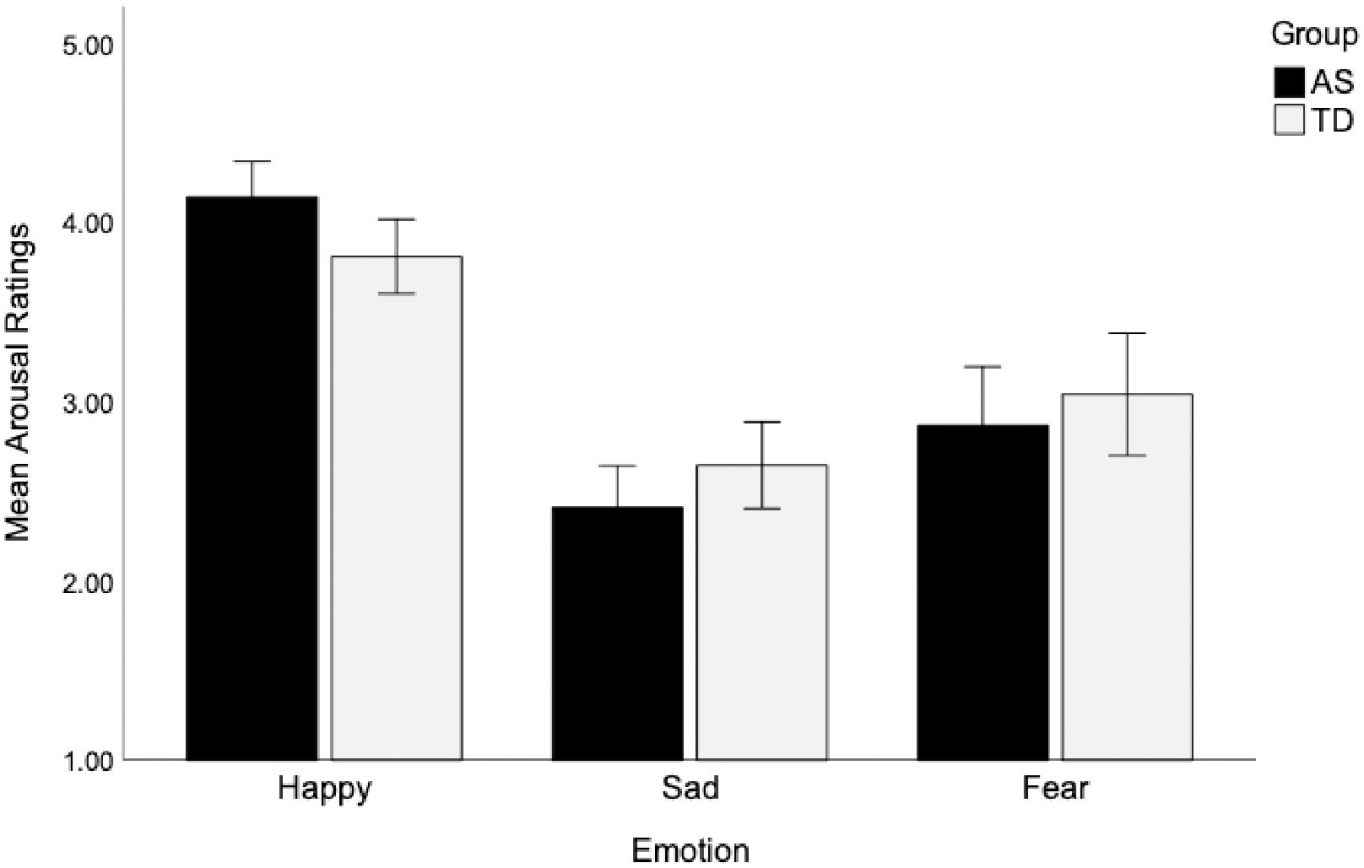
No group differences in arousal ratings for emotions. Both AS and TD groups rated happy emotions as more arousing than fear, and fear more arousing than sad. Means and standard error bars are shown.

*Arousal ratings*. A three-way interaction among group, stimulus type, and emotion on arousal ratings was not statistically significant, F(4, 180) = 1.01, *p* = .40, η_p_^2^ = .022. Further, the two-way interactions between group and stimulus type, F(2, 90) = 2.47, *p* = .09, η_p_^2^ = .052, and group and emotion, F(2, 90) = 1.87, *p* = .17, η_p_^2^ = .04, were not significant. These results suggest that the children on the AS and the TD children showed comparable patterns of arousal ratings across emotions and stimulus types (see Table 2 and Fig 4).

A significant main effect of emotion on arousal ratings was found overall, F(1.65, 74.22) = 13.97, *p* < .001, η_p_^2^ = .24, and within each group separately (AS group: F(2, 46) = 7.71, *p* = .001, η_p_^2^ = .25; TD group: F(1.58, 33.26) = 6.63, *p* = .006, η_p_^2^ = .24). Both of the groups rated happy emotions (AS: *M* = 4.12; TD: *M* = 3.84) as more arousing than fear (AS: *M* = 2.97; TD: *M* = 2.94), and fear as more arousing than sad (AS: *M* = 2.65; TD: *M* = 2.62), all *p* values ≤.005, after controlling for VMA. Neither the main effects of group and stimulus type, nor the interaction effect of stimulus type by emotion were significant (*p* values >.05), suggesting that the arousal ratings of emotions for children in both groups were equally well identified regardless of the stimulus type in which they were presented.

Although VMA was not a significant covariate in the overall model (*p* = .14), significant interactions were found between VMA and emotion, F(1.65, 74.22) = 4.96, *p* = .014, η_p_^2^ = .099, and between VMA and stimulus type, F(2, 90) = 3.33, *p* = .04, η_p_^2^ = .069. One-tailed Pearson correlations revealed that VMA was positively correlated with arousal ratings of fear (*r* = .37, *p* = .005), such that children with higher VMA rated fearful emotions as more arousing than those with lower VMA (Bonferroni corrected *p* = .0167 to account for multiple comparisons). No other correlations with emotion or stimulus type reached significance. These results suggested that VMA may have contributed to patterns of arousal ratings for particular emotions (i.e., fear).

## Discussion

Our primary objective in this study was to reconcile contrasting findings in the literature on emotion recognition (ER) among persons on the AS that include purported challenges or “deficits” in basic ER with socially explicit stimuli (primarily faces and voices) and relative strengths in ER with musical stimuli. We aimed to determine whether relative ER strengths among children on the AS were specific to music, or whether assumptions of difficulty associated with facial and vocal ER needed re-examination methodologically, via a direct group comparison of performance on a traditional categorical behavioral ER task involving musical, facial, and vocal stimuli. Music afforded a unique opportunity to compare ER skills from music to conventional ER paradigms, as musical stimuli can convey emotions without reliance on the usual interpersonal social context. Additionally, we attempted to enhance our understanding of the abilities and styles of emotion processing among children on the AS by also applying a nontraditional dimensional approach to our multi-stimulus investigation of ER. As such, our secondary objective was to take an exploratory look at patterns of performance between and within the groups using continuous ratings of valence and arousal among music, faces, and voices.

Based on the literature related to our first objective, we offered data-driven hypotheses that children on the AS as compared to TD children would demonstrate better accuracy with musical ER, and reduced accuracy with facial and vocal ER. We also hypothesized that within the groups, the children on the AS would demonstrate a strength with musical ER as compared to facial and vocal ER, whereas we expected that the TD children would perform equally well regardless of stimulus type. Given the exploratory nature of our secondary objective, we did not offer specific hypotheses about patterns of responding using dimensional valence and arousal ratings between the groups. To test our hypotheses, we developed an integrated paradigm to examine ER skills with musical, facial, and vocal stimuli conveying happy, sad, and fear emotions, using both categorical and dimensional response options concurrently.

### Patterns of basic ER among children on the AS and TD children

Within the categorical condition, we found that the children on the AS demonstrated greater accuracy in musical ER compared to the TD children, when controlling for verbal mental age (VMA), thereby supporting findings of strengths in musical ER among children on the AS [8–10]. However, in contrast with prior meta-analytic findings [6, 7, 31], the children on the AS and the TD children identified emotions equally well when using the facial and the vocal stimuli. Further, patterns of within-group performance revealed that children in both groups demonstrated the greatest ER accuracy with vocal stimuli, but that the children on the AS demonstrated comparable ER accuracy between faces and music whereas the TD children showed reduced ER accuracy with music as compared to with faces. Within the dimensional condition, the groups also performed comparably, with one exception. In terms of valence ratings, the children on the AS rated happy emotions as more positive than the TD children, but otherwise the groups rated the valence of emotions similarly across the three stimulus types. Further, both groups displayed similar patterns of arousal ratings for the three emotions and across the three stimulus types. VMA did not appear to add any further influence to our findings except for being positively correlated with arousal ratings of fear.

Considering possible explanations for our pattern of findings that stand in contrast with much of the previous ER literature necessitates a relevant discussion of methodological issues, especially as null results were found. Although we found clear discrepancies between the groups on the musical ER task, in which the AS group outperformed the TD group, our tasks may not have been sensitive enough to detect meaningful differences between the groups, thus resulting in comparable ER accuracy patterns for faces and voices. Specifically with regard to our vocal ER task, we used the most basic measure of vocal ER involving short, non-verbal vocalizations (i.e., cries, screams, and laughs) rather than verbal vocalizations which require processing of linguistic aspects of semantic content (e.g., “I am afraid”) or prosody of speech (e.g., “That’s a big shark!” said in a fearful tone), in order to understand the emotion being conveyed. Although minimizing the verbal component of the task provided confidence that ER from voices was not disproportionately reliant on participants’ verbal skills, it may have contributed to high performance accuracy overall. Nonetheless, our findings of comparable performance for ER in faces and voices are consistent with studies of socially explicit ER that also account for VMA or IQ (e.g., [40, 57–59]).

The number and type of emotion choices provided also contributes to task difficulty. Our study included three basic emotions that can easily be conveyed in music, as well as in faces and voices (and thus offered a 33% chance of randomly guessing the correct answer), with a single positive emotion and two negative emotions. Group differences may be more readily apparent when measuring complex or higher order emotion processing (e.g., [60, 61]). Further, the evidence from studies with more complex emotions and more choices (and therefore decreased accuracy attributable to chance) indicate that TD groups also perform less accurately relative to their performance with basic emotions and fewer choices [62, 63]. However, our integrated categorical and dimensional task could be extended to measure ER responses among more complex facial and vocal stimuli and to survey both basic and complex emotions (e.g., [64, 65]).

Furthermore, by design, the dimensional condition does not lend itself to constrained and categorical performance metrics, but rather allows for examination of general patterns of response using 5-point Likert scales to measure continuous components of valence and arousal. Of the 12 comparisons across two dimensions, three emotions, and three stimulus types, the only group difference was found in the perception of valence for happy emotions. The patterns of continuous valence and arousal ratings across all other emotions and stimulus types were virtually indistinguishable. Nevertheless, a lack of group differences on behavioral tasks does not necessarily exclude underlying neurophysiological differences exhibited during emotion processing. Evidence from studies of neuroimaging, skin conductance, and pupil dilation have highlighted differences in arousal levels during emotion processing tasks between TD persons and persons on the AS who also have Type II alexithymia, a difficulty with appraising and verbalizing emotions without necessarily experiencing such emotions in an atypical manner (see [52] for a review). Thus, extensions of the current study would benefit from the comparison of potential discrepancies between neurophysiological processing of emotions and the associated behavioral ratings.

Alternate ecological and sociocultural perspectives regarding why the performance of children in our study was highly accurate overall should also be considered. For example, the impact of verbal cognitive ability on ER is being increasingly considered and accounted for in research comparing children with and without developmental disabilities. Further, access to educational curricula and school-based interventions that prioritize social emotional learning (SEL), likely play a significant role in overall improvements in verbally mediated emotion learning and communication. In the United States, for example, such widespread proliferation of hundreds of universal school-based K-12 SEL programs–supported by federal educational policies–has occurred over the past few decades. Such SEL programs have led to demonstrated improvements in social and emotional skills, as well as attitudes, behaviors, and academic performance among students with a diversity of processing styles and needs [66], including those with mental health challenges such as anxiety and depression, or those with difficulty verbally expressing emotions, such as in alexithymia. Similarly, the proliferation in the past few decades of evidence-based emotion-focused interventions specifically for children on the AS that highlight the importance of identifying, expressing, and regulating emotions (e.g., PEERS [67]; SCERTS Model [68]; Facing Your Fears [69]; EASE [70]) further demonstrate increasing societal priorities surrounding the importance of research, policy, and funding for social and emotional learning for all children.

### Neuroconstructivist approaches and the case for equifinality in ER

Rather than demonstrating a singular mechanism and pattern of processing by which both groups achieve comparable task performance, our findings may instead highlight unique styles of processing for each group that lead to similar outcomes. Cicchetti and Rogosch [71] argued that the principles of multifinality and equifinality allow us to better understand possible mechanisms for observable outcomes among different populations. Whereas multifinality infers that similar developmental circumstances may lead to a heterogeneity of outcomes, equifinality suggests that various circumstances or abilities may lead to similar outcomes. The lens of equifinality specifically has been readily applied to conceptualize the heterogeneity of both genetic and behavioral characteristics of persons on the AS (i.e., multiple factors may predispose an individual to be on the AS). For example, Burack et al. [72] and others have argued that persons on the AS may engage in different styles of cognitive processing than TD persons, and thus call for greater exploration of the various ways persons on the AS engage with stimuli, rather than viewing cognitive or task performance as “deficient” or atypical compared to a reference standard. Relatedly, Johnson et al. [73, 74] have posited that the behavioral characteristics of persons on the AS may manifest as adaptive responses to early differences in neural, genetic, and experience-dependent development, and therefore impact subsequent developmental trajectories and styles of information processing. For example, differences in brain region growth or connectivity may prompt information processing reorganization (e.g., [75, 76]) in a manner that is adaptive and optimized for relevant or preferred experience-dependent learning.

The usefulness of considering neuroconstructivist approaches and applying a lens of equifinality to understand differing mechanisms of cognitive processing and ER among persons on the AS and TD persons can be highlighted in specific conceptual approaches, such as the Enhanced Perceptual Functioning (EPF) model [77, 78]. According to the EPF model, persons on the AS may have a default or preferential bottom-up approach to processing the perceptual features of a stimulus that favors local over global aspects. In this framework, global processing among persons on the AS is not necessarily impaired but may be deprioritized relative to local processing. In comparison, TD persons may favor a top-down global or holistic processing style, and may deprioritize processing at the level of local perceptual cues. Such processing differences may be an optimized or adaptive response to early developmental differences in neural structure or connectivity [79] and may be further reinforced by continued exposure and engagement with salient and motivating cues. For example, TD children spend more time looking at socially explicit stimuli (e.g., caregiver interactions, videos of social scenes) than do children on the AS [80] and therefore gain greater experience with emotional content conveyed in faces and voices. By comparison, children on the AS tend to demonstrate enhanced local processing of perceptual features of socially explicit faces [81, 82], speech [83] and audio-visual synchronous biological motion [84], in addition to their enhanced perceptual processing of less explicitly social stimuli, such as music [79]. Further, processing of emotional information through preferred or engaging stimuli such as music, which activates reward-based regions of the brain [13, 29], may thus become more salient and reinforcing–and thus, further optimized–over time.

Across all three stimulus types, basic emotions can be decoded using both top down and bottom up approaches, and thus both groups of children in our study may have been successful at decoding emotions by using their preferential styles of processing in order to categorize emotions similarly on our task. Bottom-up processing of music may confer an additional advantage to children on the AS, who show strengths in decoding perceptual musical cues (i.e., pitch, tone, energy) (e.g., [11, 12]). Thus, compared to TD children, music may readily invoke the enhanced perceptual processing styles of children on the AS and be a privileged means for conveying emotions for them.

### Implications

For a characteristic or challenge to be considered a core “deficit” primary to any particular group, it should be specific to and universal within that group, and should be one of the most persistent or impairing features [40, 85]. Overall, our findings are inconsistent with the notion that children on the AS demonstrate a core “deficit” in basic ER, and we attempt to delineate this through our findings in several ways. In terms of specificity, using a child-friendly, multi-response paradigm designed to minimize reliance on verbal cues, we found typical ER performance across the facial and vocal accuracy tasks, and comparable ratings of emotional valence and arousal, demonstrating indisputable basic ER capabilities of children on the AS. The notion of the universality of ER differences was also not borne out, given our findings of relative strength of children on the AS in musical ER, which ultimately underscore that discussions of ER broadly need not be restricted to the traditional facial or vocally expressed emotions.

The musical ER strengths highlighted in our study support the growing evidence of the benefits of music therapy for children on the AS [27]. Our findings contribute to the notion that musical strengths, including typical or enhanced processing of music-evoked emotions, may be a key component of the success of music programs and therapies that target social communication and interaction skills [23, 26–28] and speech and language interventions that incorporate music [22, 24, 25]. Music therapy, education, and interventions may be successful because they leverage emotional and cognitive strengths [13] and are motivating and accessible to children on the AS because they readily engage with and enjoy music [86, 87].

## Conclusion

The findings from this study of ER across multiple basic emotions, stimulus types, and response options, help to dispel notions of deficits of the ability to explicitly recognize and label basic emotions of children on the AS relative to TD children. Instead, findings of comparable ER accuracy (in faces and voices) or relative strengths (in musical ER) depending on the stimulus type provides support for the use of music therapy, education, and interventions. Ultimately, our findings contribute to efforts to provide a nuanced understanding of the ways in which we can re-shape our thinking about persons on the AS by engaging their preferences and strengths.

## Supporting information

S1 Table Family demographics for the AS and TD groups. Family demographics data were available for 36 of 48 participants. *p values of Fisher’s exact tests between the AS and TD groups were not significant following Bonferroni corrections for multiple comparisons.
(DOCX)
