# Supplementary material for: Basic emotion recognition of children on the autism spectrum is enhanced in music and typical for faces and voices
Source: PLoS One. 2023 Jan 11;18(1):e0279002. doi: 10.1371/journal.pone.0279002 (PMC9833514; doi:10.1371/journal.pone.0279002)
Supplement: S1 Table — Family demographics data were available for 36 of 48 participants. *p values of Fisher’s exact tests between the AS and TD groups were not significant following Bonferroni corrections for multiple comparisons. (DOCX) [file pone.0279002.s001.docx]

**S1 Table. Family Demographics for the AS and TD Groups.** Family demographics data were available for 36 of 48 participants. *p values of Fisher’s exact tests between the AS and TD groups were not significant following Bonferroni corrections for multiple comparisons.

|  | Total Sample  (*N* = 36) | AS Group  (*n* = 15) | TD Group  (*n* = 23) | *p* |
| --- | --- | --- | --- | --- |
| Maternal Education, n (%)  High School  College  Undergraduate  Post-Graduate  Other | 5 (13.9%)  7 (19.4%)  10 (27.8%)  12 (33.3%)  2 (5.5%) | 4 (26.7%)  5 (33.3%)  3 (20%)  3 (20%)  0 (0%) | 1(4.8%)  2 (9.5%)  7 (33.5%)  9 (42.9%)  2 (9.5%) | .07* |
| Annual Income, n (%)  <$40,000  $40,000-$60,000  $60,000-$80,000  $80,000-$100,000  $100,000-$125,000  >$125,000  Prefer not to say | 2 (5.5%)  5 (13.9%)  5 (13.9%)  4 (11.1%)  6 (16.7%)  5 (13.9%)  9 (25%) | 2 (13.3%)  4 (26.7%)  0 (0%)  3 (20%)  3 (20%)  2 (13.3%)  1 (6.7%) | 0 (0%)  1 (4.8%)  5 (23.8%)  1 (4.8%)  3 (14.3%)  3 (14.3%)  8 (38%) | .013* |
